# VEGFB Promotes Myoblasts Proliferation and Differentiation through VEGFR1-PI3K/Akt Signaling Pathway

**DOI:** 10.3390/ijms222413352

**Published:** 2021-12-12

**Authors:** Mingfa Ling, Lulu Quan, Xumin Lai, Limin Lang, Fan Li, Xiaohua Yang, Yiming Fu, Shengchun Feng, Xin Yi, Canjun Zhu, Ping Gao, Xiaotong Zhu, Lina Wang, Gang Shu, Qingyan Jiang, Songbo Wang

**Affiliations:** 1 Guangdong Provincial Key Laboratory of Animal Nutrition Control, College of Animal Science, South China Agricultural University, Guangzhou 510642, China; lingmingfa1989@163.com (M.L.); quanlulu1998@163.com (L.Q.); 13600482294@163.com (X.L.); langlanglimin@163.com (L.L.); kyanzed@163.com (F.L.); yangxiaohua137@163.com (X.Y.); 20202025006@stu.scau.edu.cn (Y.F.); fsc15816996874@sina.com (S.F.); yixin19981019@sina.com (X.Y.); canjunzhu@scau.edu.cn (C.Z.); gaoping@scau.edu.cn (P.G.); xtzhu@scau.edu.cn (X.Z.); wanglina@scau.edu.cn (L.W.); shugang@scau.edu.cn (G.S.); qyjiang@scau.edu.cn (Q.J.); 2 National Engineering Research Center for the Breeding Swine Industry, South China Agricultural University, Guangzhou 510642, China

**Keywords:** VEGFB, VEGFR1, C2C12, proliferation, differentiation, PI3K/Akt

## Abstract

It has been demonstrated that vascular endothelial growth factor B (VEGFB) plays a vital role in regulating vascular biological function. However, the role of VEGFB in regulating skeletal muscle cell proliferation and differentiation remains unclear. Thus, this study aimed to investigate the effects of VEGFB on C2C12 myoblast proliferation and differentiation and to explore the underlying mechanism. For proliferation, VEGFB significantly promoted the proliferation of C2C12 myoblasts with the upregulating expression of cyclin D1 and PCNA. Meanwhile, VEGFB enhanced vascular endothelial growth factor receptor 1 (VEGFR1) expression and activated the PI3K/Akt signaling pathway in a VEGFR1-dependent manner. In addition, the knockdown of VEGFR1 and inhibition of PI3K/Akt totally abolished the promotion of C2C12 proliferation induced by VEGFB, suggesting that VEGFB promoted C2C12 myoblast proliferation through the VEGFR1-PI3K/Akt signaling pathway. Regarding differentiation, VEGFB significantly stimulated the differentiation of C2C12 myoblasts via VEGFR, with elevated expressions of MyoG and MyHC. Furthermore, the knockdown of VEGFR1 rather than NRP1 eliminated the VEGFB-stimulated C2C12 differentiation. Moreover, VEGFB activated the PI3K/Akt/mTOR signaling pathway in a VEGFR1-dependent manner. However, the inhibition of PI3K/Akt/mTOR blocked the promotion of C2C12 myoblasts differentiation induced by VEGFB, indicating the involvement of the PI3K/Akt pathway. To conclude, these findings showed that VEGFB promoted C2C12 myoblast proliferation and differentiation via the VEGFR1-PI3K/Akt signaling pathway, providing new insights into the regulation of skeletal muscle development.

## 1. Introduction

Skeletal muscle is a vital organ, which occupies 30–40% of the body. The basic functions of the skeletal musculature include facilitating locomotor activity, postural behavior, and breathing, and proper muscle formation and function are required for a healthy life [1]. Impaired or a loss of function of skeletal muscle can affect functional capacity and increase the risk of many diseases, such as diabetes mellitus [2], muscle atrophy [3], and cancer [4]. Myogenesis is a highly orchestrated process that involves myoblast proliferation, migration, and differentiation, as well as the fusion of multicellular myotubes into contractile skeletal muscle fibers [5]. Therefore, promoting the proliferation and differentiation of myoblasts and inducing myotube hypertrophy should be beneficial for muscle growth and muscle mass regulation.

During myogenesis in adult mammalian skeletal muscle, activated myogenic progenitors, called myoblasts, undergo several rounds of proliferation to increase the myogenic pool needed for muscle growth or tissue repair. Myoblast proliferation is regulated by cell cycle regulators such as Cyclin D1, Cyclin E, and cyclin-dependent kinases (CDKs) [6]. Previous studies have also shown that proliferating cell nuclear antigen (PCNA) plays an important role in DNA replication and cell cycle regulation in eukaryotic cells [7], as well as participates in regulating myoblast proliferation [8]. In addition, studies have revealed that the PI3K/Akt signaling pathway is involved in myoblast proliferation by regulating the cell-cycle proteins [8,9]. Thus, skeletal muscle growth and development can benefit from the regulation of the PI3K/Akt signaling pathway and downstream target genes.

Following proliferation, myoblasts exit the cell cycle and start to differentiate [10]. In vitro, myoblasts can be amplified by high concentrations of serum, and converting to low-serum medium induces their differentiation and fusion with adjacent cells. The myogenic regulatory factors (MRFs), which are composed of myogenic factor 5 (Myf5), myogenic determining factor (MyoD), myogenin (MyoG), and muscle-specific regulatory factor 4 (MRF4), are known to play vital roles in the process of myogenesis [5,11]. The myogenic regulatory factors such as MyoD and MyoG regulate the expression of myosin heavy chain (MyHC), a myotube-specific structural protein [12]. Meanwhile, the increase in individual myotubes and myofibers size, known as hypertrophy, causes an increase in skeletal muscle mass [13]. Muscle hypertrophy occurs due to the total rates of protein synthesis exceeding the rates of protein degradation. The PI3K/Akt/mTOR signaling pathway has been shown to control protein synthesis, and additional pathways have recently been identified [3]. There are different kinds of factors playing a regulatory role in the process of skeletal muscle development, such as growth factors [14], sex hormones [15], and nutrients [16]. It has been verified that some of the vascular endothelial growth factors and receptors are involved in the process of muscle growth or muscle repair [17,18,19].

Vascular endothelial growth factor B (VEGFB) is the third vascular endothelial growth factor family member to be discovered in 1996 [20]. Being a close homolog, VEGFB is expected to have similar and redundant functions to VEGFA and PLGF during angiogenesis. However, VEGFB is not upregulated by hypoxia, is poorly angiogenic, and does not induce vascular permeability in animals or tissues, arguing against the angiogenic activity of this molecule [21,22,23]. VEGFB binds to vascular endothelial growth factor receptor 1 (VEGFR1) and neuropilin 1 (NRP1), which activates a lot of downstream activators similar to most tyrosine kinase receptors such as p38 MAPK, ERK/MAPK, PKB/AKT, and PI3K [24]. Given the controversial role of VEGFB in angiogenesis, recent studies have recognized the important role of this growth factor in cell survival in genetic studies in vivo and the use of recombinant VEGFB protein in vitro [25]. It has been reported that VEGFB preserved the function of the heart by the inhibition of apoptosis and promotion of cardiomyocyte proliferation [18,19]. In addition, both human and mouse data indicate that VEGFB has a vital role in endothelial free fatty acids uptake, via VEGFR1, NRP1, FATP3, and FATP4 [26,27,28]. However, the biological role of VEGFB has long been enigmatic, and the role of VEGFB in skeletal muscle development remains unclear.

Thus, the present study was conducted to investigate the effects of VEGFB on myoblast proliferation and differentiation. In addition, we sought to explore the potential mechanism of this process, including the role of VEGFR1 and the related intracellular signaling pathway. Our data showed that VEGFB promotes myoblasts proliferation and differentiation via the VEGFR1-PI3K/Akt signaling pathway.

## 2. Results

### 2.1. VEGFB Promoted the Proliferation of C2C12 Cells via VEGFRs Signaling

**Figure 1:**
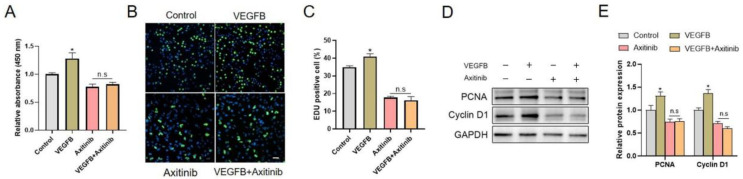
VEGFB promoted the proliferation of C2C12 cells via VEGFRs signaling. (**A**) Effect of 100 ng/mL of VEGFB and/or 2 µM of axitinib on the proliferation of C2C12 after 48 h culture was determined by CCK8 analysis (*n* = 6). (**B**) Effects of 100 ng/mL of VEGFB and/or 2 µM of axitinib on C2C12 proliferation were assessed by using EdU incorporation assay, with the EdU-positive nuclei shown in green. The nuclei were stained with Hoechst, and the scale bar = 200 µm (*n* = 3). (**C**) Percentage of EdU-positive cells in panel B. (**D**) Western blot of PCNA and cyclin D1 in C2C12 after 48 h culture. GAPDH was used as loading control. (**E**) Mean ± SEM of immunoblotting bands of PCNA and cyclin D1; the results are expressed as arbitrary units (*n* = 6). * *p* < 0.05 versus control group. n.s = not significant.

We first assessed the effect of VEGFB on the proliferation of C2C12 myoblasts. The CCK8 assay revealed that 100 ng/mL of VEGFB significantly stimulated C2C12 proliferation (Figure 1A). Meanwhile, an EdU incorporation assay demonstrated that VEGFB markedly increased the percentage of EdU-positive cells, indicating the proliferative effects of VEGFB (Figure 1B,C). However, the promotive effects of VEGFB on C2C12 proliferation was abolished by axitinib, an inhibitor of VEGFRs (Figure 1A–C). Consistent with the proliferation phenotype, the protein expression of proliferative markers such as PCNA and Cyclin D1 was elevated by VEGFB, while axitinib eliminated the VEGFB-induced increase in proliferative markers expression (Figure 1D,E). These data showed that VEGFB promoted the proliferation of C2C12 by increasing the expression of proliferative markers via VEGFRs signaling.

### 2.2. VEGFR1, but Not NRP1, Was Involved in VEGFB-Promoted Proliferation of C2C12 Cells

**Figure 2:**
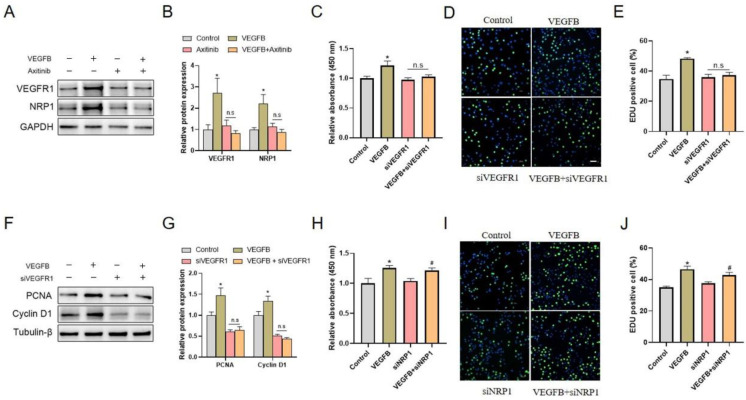
Knockdown of VEGFR1, but not NRP1, eliminated the promotion of C2C12 proliferation induced by VEGFB. (**A**) Western blot of VEGFR1 and NRP1 in C2C12 after 48 h culture. GAPDH was used as loading control. (**B**) Mean ± SEM of immunoblotting bands of VEGFR1 and NRP1; the results are expressed as arbitrary units (*n* = 6). (**C**) Effect of 100 ng/mL of VEGFB and/or siVEGFR1 on the proliferation of C2C12 after 48 h culture was determined by CCK8 analysis (*n* = 6). (**D**) Effects of 100 ng/mL of VEGFB and/or siVEGFR1 on C2C12 proliferation were assessed by using EdU incorporation assay (*n* = 3). The nuclei were stained with Hoechst. Scale bar = 200 µm. (**E**) Percentage of EdU-positive cells in panel D. (**F**) Western blot analysis of PCNA and cyclin D1 in C2C12 after 48 h culture. β-Tubulin was used as loading control. (**G**) Mean ± SEM of immunoblotting bands of PCNA and cyclin D1; the results are expressed as arbitrary units (*n* = 3). (**H**) Effect of 100 ng/mL of VEGFB and/or siNRP1 on the proliferation of C2C12 after 48 h culture was determined by CCK8 analysis (*n* = 6). (**I**) Effects of 100 ng/mL of VEGFB and/or siNRP1 on C2C12 proliferation by using EdU incorporation assay (*n* = 3). The nuclei were stained with Hoechst. Scale bar = 200 µm. (**J**) Percentage of EdU-positive cells in panel H. * *p* < 0.05 versus control group. # *p* < 0.05 versus the siNRP1 group. n.s = not significant. siVEGFR1 and siNRP1, small interfering RNA for VEGFR1 and NRP1, respectively.

As VEGFB binds to the receptors VEGFR1 and NRP1, we further determined the contribution of VEGFR1 and NRP1 in the VEGFB-promoted proliferation of C2C12 cells. We detected the expression of VEGFR1 and NRP1 in response to VEGFB and found that VEGFB significantly increased the protein expression of VEGFR1 and NRP1 (Figure 2A,B). The similar pattern between the promotion of C2C12 proliferation and enhancement of VEGFR1 and NRP1 expression suggested that VEGFR1 and NRP1 might be involved in VEGFB-stimulated C2C12 proliferation. In addition, the knockdown of VEGFR1 with VEGFR1 siRNA abolished the promotion of C2C12 proliferation (Figure 2C) and the increase in EdU-positive cells induced by VEGFB (Figure 2D,E). Accordingly, the increased protein expression of PCNA and cyclin D1 induced by VEGFB was eliminated by VEGFR1 siRNA (Figure 2F,G). By contrast, the knockdown of NRP1 with NRP1 siRNA did not reverse the promotion of C2C12 proliferation (Figure 2H) and the elevation of EdU-positive cells (Figure 2I,J) induced by VEGFB. Together, these findings revealed that VEGFR1, but not NRP1, was involved in the VEGFB-promoted proliferation of C2C12 cells, indicating the significant role of VEGFR1 in this process.

### 2.3. PI3K/Akt Signaling Pathway Was Involved in C2C12 Proliferation Induced by VEGFB

**Figure 3:**
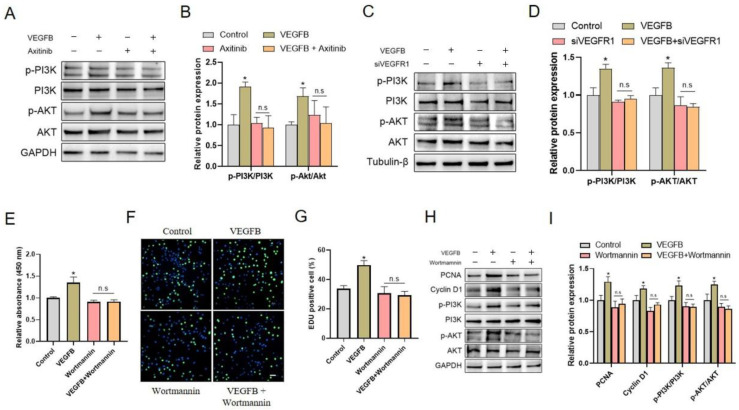
VEGFB activated the PI3K/Akt signaling pathway in a VEGFR1-dependent manner, and the inhibition of PI3K/Akt blocked the promotion of C2C12 proliferation induced by VEGFB. (**A**) Western blot analysis of p-PI3K, PI3K, p-Akt, and Akt in C2C12 after 48 h culture. GAPDH was used as loading control. (**B**) Mean ± SEM of immunoblotting bands of p-PI3K, PI3K, p-Akt, and Akt in panel A (*n* = 3). (**C**) Western blot of p-PI3K, PI3K, p-Akt, and Akt in C2C12 after 48 h culture. β-Tubulin was used as loading control. (**D**) Mean ± SEM of immunoblotting bands of p-PI3K, PI3K, p-Akt, and Akt in panel C; the results are expressed as arbitrary units (*n* = 3). (**E**) Effect of 100 ng/mL of VEGFB and/or 2 µM of Wortmannin on the proliferation of C2C12 after 48 h culture was determined by CCK8 analysis (*n* = 6). (**F**) Effects of 100 ng/mL of VEGFB and/or 2 µM of Wortmannin on C2C12 proliferation were assessed by using EdU incorporation assay (n = 3). The nuclei were stained with Hoechst, and the scale bar = 200 µm. (**G**) Percentage of EdU-positive cells in panel F. (**H**) Western blot analysis of PCNA, cyclin D1, p-PI3K, PI3K, p-Akt, and Akt in C2C12 after 48 h culture. GAPDH was used as loading control. (**I**) Mean ± SEM of immunoblotting bands of PCNA, cyclin D1, p-PI3K, PI3K, p-Akt, and Akt; the results are expressed as arbitrary units (*n* = 3). * *p* < 0.05 versus control group. n.s = not significant. siVEGFR1, small interfering RNA for VEGFR1.

We further assessed the potential involvement of the intracellular PI3K/Akt signaling pathway in VEGFB-induced C2C12 proliferation. The results showed that VEGFB significantly increased the ratios of p-PI3K/PI3K and p-Akt/Akt, indicating the activation of the PI3K/Akt signaling pathway. Interestingly, the activation of the PI3K/Akt signaling pathway induced by VEGFB was eliminated by VEGFR inhibitor axitinib (Figure 3A,B), and VEGFR1 siRNA (Figure 3C,D), implying a link between VEGFR1 and PI3K/Akt activation. These data indicated that VEGFB activated the PI3K/Akt signaling pathway in a VEGFR1-dependent manner and suggested that the activation of the intracellular PI3K/Akt signaling pathway might be involved in VEGFB-stimulated C2C12 proliferation.

To further verify the role of the PI3K/Akt signaling pathway in the VEGFB-stimulated proliferation of C2C12 cells, Wortmannin, an inhibitor of PI3K, and siPIK3CA, a siRNA target to PI3KCA, were used to inhibit the activation of PI3K/Akt signaling in this study. Indeed, the increases in p-PI3K/PI3K and p-Akt/Akt ratio in response to VEGFB were reversed by Wortmannin (Figure 3H,I) and siPIK3CA (Figure S1D,E). Importantly, the results demonstrated that Wortmannin and siPIK3CA blocked the promotion of C2C12 proliferation (Figure 3E, Figure S1A) and the increase in EdU-positive cells (Figure 3F,G, Figure S1B,C) induced by VEGFB. In agreement, the enhanced protein expressions of PCNA and cyclin D1 induced by VEGFB were abolished by Wortmannin (Figure 3H,I) and siPIK3CA (Figure S1D,E). These results strongly suggested that the activation of the PI3K/Akt signaling pathway was involved in VEGFB-promoted C2C12 proliferation.

### 2.4. VEGFB Stimulated the Differentiation of C2C12 Cells through VEGFRs Signaling

**Figure 4:**
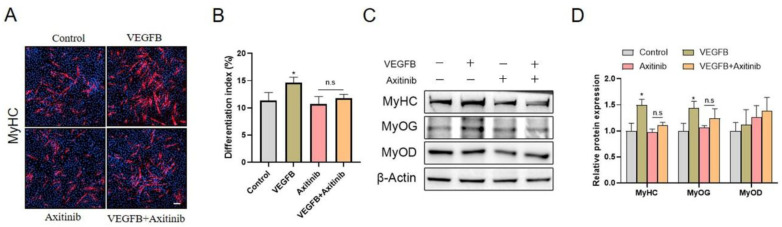
VEGFB stimulated the differentiation of C2C12 cells via VEGFRs signaling. (**A**) Effect of 100 ng/mL of VEGFB and/or Axitinib on the differentiation of C2C12 after 5 days of differentiation was determined by immunofluorescence of MyHC (*n* = 3). The nuclei were stained with Hoechst, and the scale bar = 200 µm. (**B**) The differentiation index was counted by comparing the MyHC-positive cells to total nuclei. (**C**) Western blot analysis of MyHC, MyoD, and MyoG in C2C12 after 5 days of differentiation. β-Actin was used as loading control. (**D**) Mean ± SEM of immunoblotting bands of MyHC, MyoD, and MyoG; the results are expressed as arbitrary units (*n* = 6). * *p* < 0.05 versus control group. n.s = not significant.

The effects of VEGFB on the differentiation of C2C12 cells were further evaluated. The morphological changes of the C2C12 myoblast differentiation were observed by using immunofluorescence staining of myosin heavy chain (MyHC). The results demonstrated that the numbers of multinucleated myotubes and the differentiation index were significantly increased in response to VEGFB treatment, suggesting the stimulation of C2C12 differentiation by VEGFB (Figure 4). However, the VEGFB-induced promotion of C2C12 differentiation was abolished by VEGFR inhibitor axitinib (Figure 4A,B). Consistently, the protein expression of MyHC and myogenin (MyoG) was enhanced by VEGFB, while the VEGFB-induced enhancement of MyHC and MyoG expression was eliminated by axitinib (Figure 4C,D). Taken together, these data suggested that VEGFB stimulated the differentiation of C2C12 through VEGFRs signaling.

### 2.5. Knockdown of VEGFR1, Rather Than NRP1, Eliminated the Stimulation of C2C12 Differentiation Induced by VEGFB

**Figure 5:**
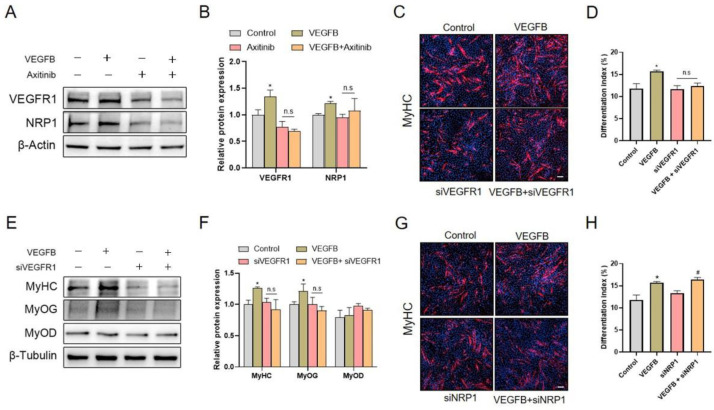
Knockdown of VEGFR1, rather than NRP1, was involved in VEGFB-promoted differentiation of C2C12 cells. (**A**) Western blot analysis of VEGFR1 and NRP1 in C2C12 after 5 days of differentiation. β-Actin was used as loading control. (**B**) Mean ± SEM of immunoblotting bands of VEGFR1 and NRP1; the results are expressed as arbitrary units (*n* = 6). (**C**) Effect of 100 ng/mL of VEGFB and/or siVEGFR1 on the differentiation of C2C12 after 5 days of differentiation was determined by immunofluorescence of MyHC (*n* = 3). The nuclei were stained with Hoechst, and the scale bar = 200 µm. (**D**) The differentiation index was counted by comparing the MyHC-positive cells to total nuclei in panel C. (**E**) Western blot analysis of MyHC, MyoD, and MyoG in C2C12 after 5 days of differentiation. β-Tubulin was used as loading control. (**F**) Mean ± SEM of immunoblotting bands of MyHC, MyoD, and MyoG; the results are expressed as arbitrary units (*n* = 3). (**G**) Effect of 100 ng/mL of VEGFB and/or siNRP1 on the differentiation of C2C12 after 5 days of differentiation was determined by immunofluorescence of MyHC (*n* = 3). Scale bar = 200 µm (**H**) The differentiation index was counted by comparing the MyHC-positive cells to total nuclei in panel G. The nuclei were stained with Hoechst; * *p* < 0.05 versus control group. # *p* < 0.05 versus the siNRP1 group. n.s = not significant. siVEGFR1 and siNRP1, small interfering RNA for VEGFR1 and NRP1, respectively.

We further examined the effects of VEGFB on the expression of VEGFR1 and NRP1 and observed that VEGFB significantly increased the protein expressions of VEGFR1 and NRP1 (Figure 5A,B). These results suggested that VEGFR1 and NRP1 might participate in VEGFB-stimulated C2C12 differentiation. To further verify the role of VEGFR1 and NRP1 in VEGFB-stimulated C2C12 differentiation, VEGFR1 and NRP1 siRNA were applied to knock-down the expression of VEGFR1 and NRP1 in the present study. Intriguingly, we found that the knockdown of VEGFR1 with VEGFR1 siRNA abolished the stimulation of C2C12 differentiation and myotube formation induced by VEGFB (Figure 5C,D). Consistently, the increased protein expressions of MyHC and MyoG induced by VEGFB were eliminated by VEGFR1 siRNA (Figure 5E,F). Unlike the role of VEGFR1, the knockdown of NRP1 with NRP1 siRNA could not reverse the promotion of C2C12 differentiation induced by VEGFB (Figure 5G,H). Overall, these results demonstrated that the knockdown of VEGFR1, rather than NRP1, eliminated the stimulatory effects of VEGFB on C2C12 differentiation, thereby indicating the essential role of VEGFR1 in C2C12 differentiation.

### 2.6. PI3K/AKT/mTOR Signaling Pathway Was Involved in VEGFB-Stimulated C2C12 Differentiation

**Figure 6:**
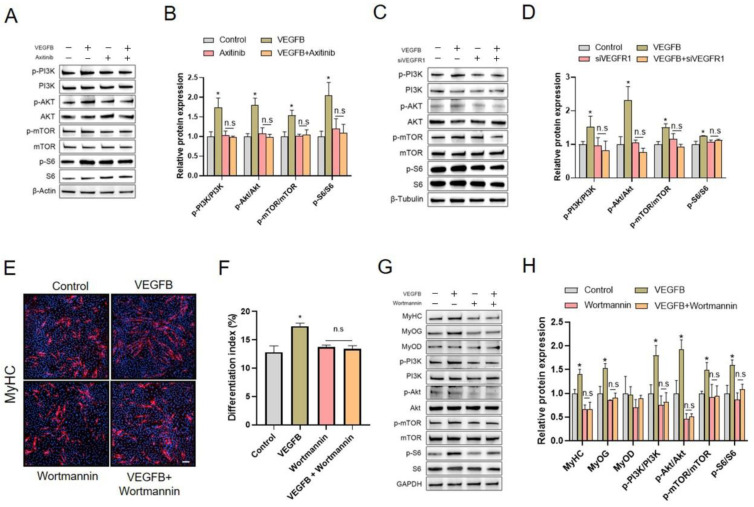
VEGFB activated the PI3K/Akt/mTOR signaling pathway in a VEGFR1-dependent manner and the inhibition of PI3K/Akt blocked the promotion of C2C12 differentiation induced by VEGFB. (**A**) Western blot analysis of p-PI3K, PI3K, p-Akt, Akt p-mTOR, mTOR, p-S6, and S6 in C2C12 treated with or without VEGFB and/or axitinib after 5 days of differentiation. (**B**) Mean ± SEM of immunoblotting bands of p-PI3K/PI3K, p-Akt/Akt p-mTOR/mTOR, and p-S6/S6 in panel A. (**C**) Western blot analysis of p-PI3K, PI3K, p-Akt, Akt p-mTOR, mTOR, p-S6, and S6 in C2C12 treated with or without VEGFB and/or siVEGFR1 after 5 days of differentiation. (**D**) Mean ± SEM of immunoblotting bands of p-PI3K/PI3K, p-Akt/Akt, p-mTOR/mTOR, and p-S6/S6 in panel C; the results are expressed as arbitrary units (*n* = 3). (**E**) Effect of 100 ng/mL of VEGFB and/or 2 µM of Wortmannin on the differentiation of C2C12 after 5 days of differentiation was determined by immunofluorescence of MyHC (*n* = 3). The nuclei were stained with Hoechst and the scale bar = 200 µm. (**F**) The differentiation index was counted by comparing the MyHC-positive cells to total nuclei in panel E. (**G**) Western blot analysis of MyHC, MyoD, MyoG, p-PI3K/PI3K, p-Akt/Akt, p-mTOR/mTOR, and p-S6/S6 in C2C12 after 5 days of differentiation. GAPDH was used as loading control. (**H**) Mean ± SEM of immunoblotting bands of MyHC, MyoD, MyoG, p-PI3K/PI3K, p-Akt/Akt, p-mTOR/mTOR, and p-S6/S6; the results are expressed as arbitrary units (*n* = 3). * *p* < 0.05 versus control group. n.s = not significant. siVEGFR1, small interfering RNA for VEGFR1.

We further explored the possible intracellular signaling pathway in VEGFB-stimulated C2C12 differentiation. The results showed that VEGFB significantly increased the ratio of p-PI3K/PI3K, p-Akt/Akt, p-mTOR/mTOR, and p-S6/S6, indicating the activation of the PI3K/Akt/mTOR signaling pathway (Figure 6). Interestingly, the activation of the PI3K/Akt/mTOR signaling pathway induced by VEGFB was eliminated by VEGFRs inhibitor axitinib (Figure 6A,B) and VEGFR1 siRNA (Figure 6C,D), indicating a link between VEGFR1 and PI3K/Akt/mTOR signaling activation. These results suggested that VEGFB activated the PI3K/Akt/mTOR signaling pathway in a VEGFR1-dependent manner and implied the possible involvement of the PI3K/Akt/mTOR signaling pathway in VEGFB-stimulated C2C12 differentiation. 

To further verify the role of the PI3K/Akt/mTOR signaling pathway in the VEGFB-stimulated differentiation of C2C12, Wortmannin and siPIK3CA were applied to inhibit the activation of PI3K and thus to prevent the activation of Akt and mTOR in this study. As expected, the increases in p-PI3K/PI3K, p-Akt/Akt, p-mTOR/mTOR, and p-S6/S6 ratio in response to VEGFB were eliminated by Wortmannin (Figure 6G,H) and siPIK3CA (Figure S2C,D). In addition, we observed that both Wortmannin and siPIK3CA abolished the stimulation of C2C12 differentiation induced by VEGFB (Figure 6E,F, Figure S2A,B). In good agreement, the VEGFB-induced increase in the protein expressions of MyHC and MyoG was blocked by Wortmannin (Figure 6G,H) and siPIK3CA (Figure S2C,D). Taken together, these findings strongly suggested that the PI3K/AKT/mTOR signaling pathway was involved in VEGFB-stimulated C2C12 differentiation.

## 3. Discussion

In the present study, we determined that VEGFB promotes myoblasts proliferation and differentiation via the VEGFR1-PI3K/Akt signaling pathway. Myoblast proliferation and differentiation make great contributions to muscle development and repair, which can be mediated by a variety of factors, including growth factors, hormones, and nutrition [29,30,31]. To date, it has been revealed that VEGFB play a vital role in regulating the proliferation and differentiation of different kinds of cell types. Rasanen et al. demonstrated that the myocardial VEGFB transgene promoted the formation of endocardium-derived coronary vessels during development by increasing endothelial proliferation [32]. Huusko et al. also reported that the VEGFB transgene plays a vital role in regulating cardiomyocytes proliferation [18]. Consistent with previous studies, our results demonstrated that VEGFB promoted C2C12 proliferation, with increased cell viability and the percentage of DNA replication cells, as well as the expression of proliferative markers such as Cyclin D1 and PCNA. Meanwhile, we found that VEGFB increased the expression of MyHC and MyoG and the ratio of MyHC-positive cells to total nuclei, called differentiation index, in a model of differentiation of skeletal muscle cells in vitro, which indicated that VEGFB promoted C2C12 differentiation. Similarly, a recent study implied that VEGFB was involved in adipocyte differentiation [33]. Collectively, our data showed, for the first time, that VEGFB was involved in skeletal muscle cell proliferation and differentiation in vitro.

As VEGFB binds to VEGFR1 and NRP1, we further investigated the possible role of VEGFR1 and NRP1 in VEGFB-induced C2C12 proliferation and differentiation. We found that the expressions of VEGFR1 and NRP1 were both enhanced by VEGFB in the proliferation and differentiation subjects, implying the possible involvement of VEGFR1 and NRP1 in C2C12 myoblast proliferation and differentiation. Moreover, the protein expressions of VEGFR1 and NRP1 increased by VEGFB were eliminated by axitinib, an inhibitor of VEGFRs. However, the knockdown of VEGFR1 rather than NRP1 eliminated the VEGFB-stimulated C2C12 proliferation and differentiation, indicating that VEGFR1 rather than NRP1 was involved in the VEGFB-induced C2C12 myoblast proliferation and differentiation. Together, our results showed that VEGFR1 but not NRP1 was responsible for the promotive effects of VEGFB on C2C12 myoblast proliferation and differentiation. Similarly, Li et al. demonstrated that VEGFB inhibited apoptosis via VEGFR1-mediated inhibition of the expression of BH3-only protein genes in mice and rats [34]. However, Jensen et al. reported that VEGFB-neuropilin 1 signaling was indispensable for vascular development in zebrafish [35]. Although VEGFR1 and NRP1 are both expressed in the C2C12 myoblast, the different selections and biological functions of VEGFB binding to VEGFR1 or NRP1 might be due to the different species, cell types, and treatment systems.

It has been demonstrated that VEGFB binding to VEGFR1 leads to activation of a number of downstream activators similar to most tyrosine kinase receptors, including the PI3K/Akt signaling pathway [24]. A lot of studies have demonstrated that the activation of the PI3K/Akt signaling pathway was involved in regulating the proliferation of stem cells [36], cancer cells [37], and fibroblasts [38], as well as artery smooth muscle cells [39]. In line with the previous studies, we found that the PI3K/Akt signaling pathway was activated by VEGFB during C2C12 proliferation, and the activation of PI3K/Akt was abolished by axitinib and VEGFR1 knockdown. Moreover, the inhibition of the PI3K/Akt signaling pathway with Wortmannin reversed the VEGFB-induced stimulation of C2C12 proliferation, increased the cell viability and EdU-positive cells, and increased the expression of proliferative markers. These results demonstrated that VEGFB stimulated C2C12 proliferation via the activation of VEGFR1 and the linked intracellular PI3K/Akt signaling pathway.

Muscle hypertrophy makes great contributions to skeletal muscle growth with an increase in skeletal muscle mass. Muscle hypertrophy occurs by the fusion of myoblasts into multicellular myotubes, which was strongly controlled by the PI3K/Akt/mTOR signaling pathway [40]. Although numerous studies have demonstrated that the PI3K/Akt/mTOR signaling pathway is significant for cell growth, survival, and differentiation in skeletal muscle [41,42,43], conflicting reports have suggested different roles for the PI3K/Akt/mTOR pathway in muscle proliferation and differentiation. A previous study reported that the PI3K/Akt/mTOR pathway is a pivotal signaling pathway leading to skeletal muscle differentiation, and the inhibition of PI3K blocks the differentiation of mouse skeletal muscle cell lines [44]. Accordingly, we found that VEGFB promoted C2C12 myoblast differentiation, along with the activation of the PI3K/Akt/mTOR signaling pathway. The activation of the PI3K/Akt/mTOR signaling pathway led to elevated protein synthesis in C2C12 myoblasts. Meanwhile, we observed that the inhibition of PI3K/Akt with Wortmannin and VEGFR1 knockdown abolished C2C12 myoblast differentiation induced by VEGFB. These results suggested that VEGFB promoted myoblasts differentiation via the activation of VEGFR1 and the linked intracellular PI3K/Akt/mTOR signaling pathway. By contrast, a recent study reported that PI3K/Akt/mTOR inhibition induced oral cancer cell cycle arrest in vitro and in vivo, implying the promotive effect of the PI3K/Akt/mTOR pathway on cell proliferation [45]. These discrepant effects of PI3K/Akt/mTOR on cell proliferation and differentiation might be attributed to the different cell types and culture conditions. In addition, previous studies have demonstrated that the activation of the PI3K/Akt signaling pathway was involved in muscle cell differentiation by regulating MyoG and MyoD, the key transcription factors for muscle cell differentiation [46,47,48]. In agreement, we found that Wortmannin and VEGFR1 knockdown reversed the protein expression of MyoG, but not MyoD, induced by VEGFB, indicating that VEGFB-induced expression of the MyoG was involved in C2C12 myoblast differentiation. Moreover, the inhibition of PI3K/Akt with Wortmannin blocked the protein expression of MyoG induced by VEGFB. Collectively, these observations suggested that VEGFB promotes myoblasts differentiation via the activation of VEGFR1 and the linked intracellular PI3K/Akt signaling pathway.

**Figure 7:**
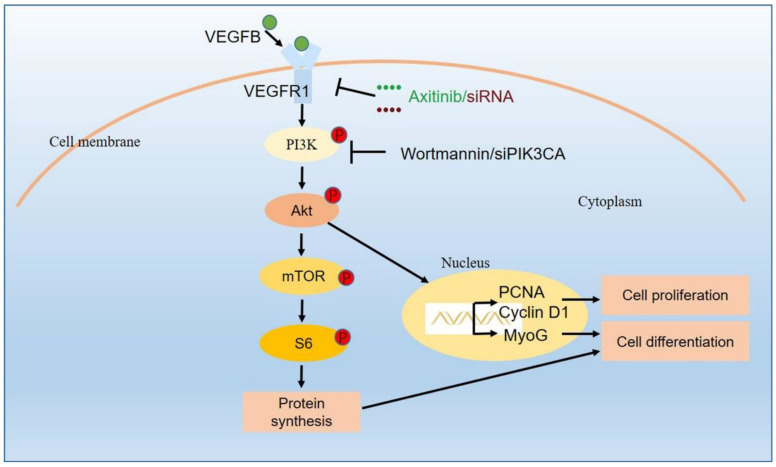
Proposed signaling pathways for VEGFB to promote C2C12 myoblasts proliferation and differentiation.

In conclusion, our findings demonstrated that VEGFB promoted myoblasts proliferation and differentiation via the VEGFR1-PI3K/Akt signaling pathway, as described in Figure 7. These results provided new insights into the regulation of skeletal muscle repair and regeneration, as well as developmental and postnatal myogenesis.

## 4. Materials and Methods

### 4.1. Materials and Reagents

Recombinant mouse VEGFB protein (#293-VE-010) was purchased from R&D systems (Minneapolis, MN, USA). High-glucose Dulbecco’s modified Eagle’s medium (DMEM-high glucose), horse serum (HS), and fetal bovine serum (FBS) were purchased from Gibco BRL (Gaithersburg, MD, USA). The Cell Count Kit 8 (CCK-8), 5-ethynyl-2′-deoxyuridine (EdU) incorporation assay kit, and DAPI were purchased from Beyotime Biotechnology Co., Ltd. (Shanghai, China). Axitinib and Wortmannin were purchased from Selleckchem (Houston, TX, USA). Lipofectamine 2000 was purchased from Life Technologies (Carlsbad, CA, USA). VEGFR1 and NRP1 siRNA were purchased from Sangon Biotechnology Co., Ltd. (Shanghai, China). PIK3CA (PI3K p110α) siRNA was purchased from Santa Cruz Biotechnology (Shanghai, China) Co., Ltd. Antibodies against PCNA and Cyclin D1 were purchased from Zen-bioscience Company (Chengdu, China). Antibodies against VEGFR1 and MyoG were purchased from Abcam (Cambridge, MA, USA). Antibodies against MyoD and NRP1 were purchased from Santa Cruz Biotechnology, Inc. (Dallas, TX, USA). Antibodies against PI3K and p-PI3K (Try317) were purchased from Beijing Bioss Biotechnology Institute (Beijing, China). Antibodies against p-Akt (Ser473), Akt, p-mTOR (Ser2448), mTOR, p-S6, and S6 were purchased from Cell Signaling Technology, Inc. (Danvers, MA, USA). Antibodies against MyHC were purchased from R&D systems (Minneapolis, MN, USA). Antibodies against β-actin, β-tubulin, and GAPDH were purchased from Bioworld technology, Co., Ltd. (Nanjing, China). The goat-anti-mouse Cy3 conjugated secondary antibodies were purchased from Beijing Bioss Biotechnology Co., Ltd. (Beijing, China). The goat anti-mouse HRP conjugated secondary antibody and goat anti-rabbit HRP conjugated secondary antibody were purchased from Bioworld Technology, Inc. (St. Louis Park, MN, USA).

### 4.2. Cell Culture and Treatments

C2C12 cells were cultured in high-glucose DMEM supplemented with 10% of FBS, 1% of penicillin (100 U/mL), and streptomycin (100 U/mL). The C2C12 cells were maintained in a humid atmosphere (5% of CO_2_, 95% of air) at 37 °C. The differentiation of the C2C12 myoblasts into myotubes was induced using a medium containing high-glucose DMEM and 2% horse serum for 5 days. The cell culture medium was changed every 2 days.

### 4.3. Cell Proliferation Assay

The cell proliferation assay was performed using a Cell Counting Kit-8 and EdU incorporation assay kit according to the manufacturer’s protocol. C2C12 myoblasts were seeded in 96-well plates and cultured in high-glucose DMEM. The optical density at 450 nm wavelength (OD450) was measured after 48 h of treatment with or without VEGFB and/or inhibitor. The EdU incorporation assay was performed using an EdU kit. Briefly, C2C12 myoblasts were seeded in 96-well plates and cultured in high-glucose DMEM for 48 h of treatment with or without VEGFB and/or inhibitors. Subsequently, cells were incubated with EdU for 2 h, fixed with 4% paraformaldehyde for 15 min, and permeated with 0.4% Triton X-100 for another 10 min at room temperature. The cells were incubated with the Click Reaction Mixture for 30 min in a dark place and then incubated with DAPI for 10 min at room temperature. A Nikon Eclipse Ti-s microscope was used to take photos of cell staining (Nikon Instruments, Tokyo, Japan). Each experiment was repeat three times and randomly selected fields more than 5 were photographed in each well. The cell proliferation of the C2C12 cells was assessed by the ratio of EdU-positive cells to DAPI cells per well.

### 4.4. Transfection of C2C12 Cells with siRNA

siRNA targets to VEGFR1, NRP1, and PIK3CA were transfected into C2C12 cells for 6 h using Lipofectamine 2000, according to the manufacturer’s instructions. A nontargeting siRNA was used as a negative control. Subsequently, the medium was switched to complete medium and the cells were incubated with or without VEGFB and/or inhibitor for 2 days and 5 days in a cell proliferation experiment and differentiation experiment, respectively.

### 4.5. Immunofluorescence Staining

After 5 days of differentiation, the cells were washed with PBS once and fixed with 4% paraformaldehyde for 30 min at room temperature. The cells were then washed with PBS 3 times for 15 min in total, incubated in blocking buffer (5% BSA in PBS) for an hour, and incubated with MyHC antibodies (1:400 dilution) overnight at 4 °C in a wet box. The cells incubated with blocking buffer served as negative controls. The cells were then washed with PBS 3 times for 15 min in total, and incubated with a fluorescent secondary antibody (1:1000 dilution in blocking buffer) for 1 h at room temperature. The cells were then washed with PBS 3 times for 15 min in total and then stained with DAPI. Images were taken with a Nikon Eclipse Ti-s microscope (Nikon Instruments, Tokyo, Japan), and randomly selected fields more than 5 were photographed in each well.

### 4.6. Western Blotting Analysis

We used RIPA lysis buffer containing 1 mM of PMSF to lyse C2C12 cells. Protein concentration was determined with a BCA protein assay kit (Thermo Fisher Scientific, Waltham, MA, USA). Fifteen to twenty micrograms of total protein was used for electrophoresis and then transferred to a polyvinylidene fluoride membrane. Primary antibodies against VEGFR1 (1:1000), NRP1 (1:1000), PCNA (1:1000), Cyclin D1 (1:1000), MyHC (1:2000), MyoG (1:2000), MyoD (1:1000), p-PI3K (1:1000), PI3K (1:2000), p-Akt (1:1000), Akt (1:2000), p-mTOR (1:1000), mTOR (1:1000), p-S6 (1:1000), and S6 (1:1000) were used in Western blot analysis. Β-actin, β-tubulin, and GAPDH (1:10,000) were selected as a loading control. Protein expression levels were determined using Adobe photoshop CS6 (Adobe, San Jose, CA, USA).

### 4.7. Statistical Analysis

Statistical analyses were carried out by using the SPSS 19.0 (IBM SPSS, Chicago, IL, USA). Data were presented as means ± SEM. *p*-values were calculated using a two-tailed unpaired Student’s *t*-test related to the indicated group. Values of *p* < 0.05 were considered statistically significant.

## Data Availability

All important data are included in the manuscript and raw data are available upon request.

## Supplementary Materials

The following are available online at https://www.mdpi.com/article/10.3390/ijms222413352/s1.
