# Supplementary material for: VEGFB Promotes Myoblasts Proliferation and Differentiation through VEGFR1-PI3K/Akt Signaling Pathway
Source: Int J Mol Sci. 2021 Dec 12;22(24):13352. doi: 10.3390/ijms222413352 (PMC8707860; doi:10.3390/ijms222413352)
Supplement: Supplementary file 1 [file ijms-22-13352-s001.zip › ijms-1487747-supplementary.pdf]

Supplementary Materials

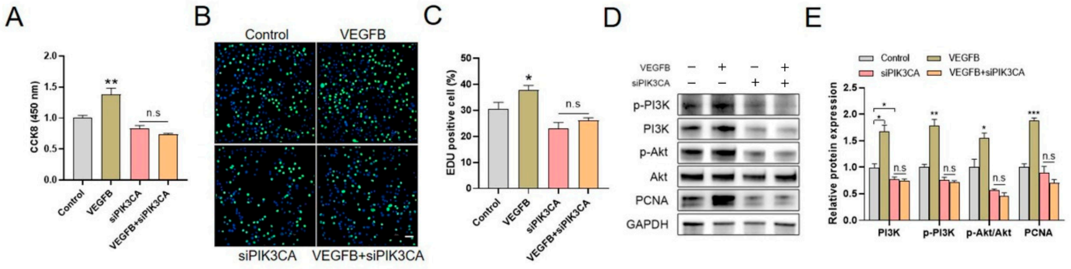

Figure S1. Inhibition of PI3K/Akt by knockdown of PIK3CA blocked the promotion of C2C12 proliferation induced by VEGFB. (A) Effect of 100 ng/mL VEGFB and/or siPIK3CA on the proliferation of C2C12 after 48 h culture was determined by CCK8 analysis (*n* = 6). (B) Effects of 100 ng/mL VEGFB and/or siPIK3CA on C2C12 proliferation were assessed by using EdU incorporation assay (*n* = 3). The nuclei were stained with Hoechst, and the scale bar = 200 μm. (C) Percentage of EdU positive cells in panel B. (D) Western blot analysis of p-PI3K, PI3K, p-Akt, Akt, and PCNA in C2C12 after 48 h culture. GAPDH was used as loading control. (E) Mean ± SEM of immunoblotting bands of p-PI3K, PI3K, p-Akt, Akt, and PCNA; the results are expressed as arbitrary units (*n* = 3). * P < 0.05, **P < 0.01, *** P < 0.001 versus control group. n.s = not significant. siPIK3CA, small interfering RNA for PIK3CA.

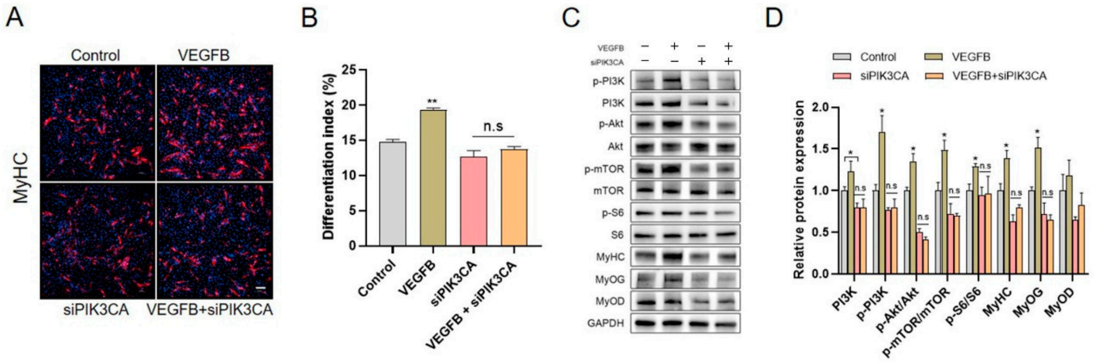

Figure S2. Inhibition of PI3K/Akt by knockdown of PIK3CA blocked the promotion of C2C12 differentiation induced by VEGFB. (A) Effect of 100 ng/mL VEGFB and/or siPIK3CA on the differentiation of C2C12 after 5 days differentiation was determined by immunofluorescence of MyHC (*n* = 3). The nuclei were stained with Hoechst and the scale bar = 200 μm. (B) The differentiation index was counted by comparing the MyHC-positive cells to total nuclei in panel A. (C) Western blot analysis of p-PI3K, PI3K, p-Akt/Akt, p-mTOR/mTOR, p-S6/S6 MyHC, MyoG, and MyoD in C2C12 after 5 days differentiation. GAPDH was used as loading control. (D) Mean ± SEM of immunoblotting bands of p-PI3K, PI3K, p-Akt/Akt, p-mTOR/mTOR, p-S6/S6 MyHC, MyoG, and MyoD; the results are expressed as arbitrary units (*n* = 3). *P < 0.05, **P < 0.01 versus control group. n.s = not significant. siPIK3CA, small interfering RNA for PIK3CA.
